# Editorial: Accelerating genetic gain for key traits using genome-wide association studies and genomic selection: promising breeding tools for sustainable agriculture

**DOI:** 10.3389/fgene.2023.1351870

**Published:** 2023-12-20

**Authors:** Dwijesh Chandra Mishra, Neeraj Budhlakoti, Philomin Juliana, Sundeep Kumar

**Affiliations:** ^1^ ICAR-Indian Agricultural Statistics Research Institute, New Delhi, India; ^2^ Borlaug Institute for South Asia (BISA), Ludhiana, India; ^3^ ICAR-National Bureau of Plant Genetic Resources, New Delhi, India

**Keywords:** GWAS, genetic gain, sustainable agriculture, genomic selection (GS), breeding programs

The pursuit of sustainable agriculture is undergoing a transformative shift through the integration of advanced genomic tools, namely, Genome-Wide Association Studies (GWAS) and Genomic Selection (GS). These tools offer a revolutionary approach to accelerating genetic gain for key traits in crop breeding programs. GWAS, by scrutinizing extensive sets of genetic markers across diverse germplasm, unveils associations between specific genomic regions and trait variations. This not only elucidates the intricate genetic factors governing desired traits but also facilitates the identification of valuable alleles for breeding programs. Complementing GWAS, Genomic Selection leverages sophisticated statistical models and high-throughput genotyping to predict the breeding value of individuals based on their entire genomic information. This predictive power enables breeders to identify superior individuals for traits of interest at an early stage, significantly expediting the breeding cycle. The synergy between GWAS and GS holds immense promise for sustainable agriculture by expediting the development of improved crop varieties, enhancing yields, stress resilience, and resource-use efficiency–crucial components of sustainable agricultural practices. Moreover, the integration of genomics into breeding programs facilitates the adaptation of crops to specific agroecological contexts and evolving climatic conditions, ensuring the resilience of crops in the face of changing climates. This editorial embarks on a nuanced journey through a selection of pioneering studies, each offering a unique lens into the multifaceted world of plant genomics through GWAS and Genomic Selection and its profound implications for the future of agriculture.

The study of *StTGA* genes in potatoes emerges as a beacon of hope in the battle against bacterial wilt, a formidable adversary threatening potato crops worldwide. *StTGA* genes, identified as a subgroup of bZIP transcription factors, play a crucial role in orchestrating the plant’s defense mechanisms against *Ralstonia solanacearum*, the causative agent of bacterial wilt. Tian et al. meticulously analyzed the expression patterns of specific *StTGA* genes during infection, unveiling a sophisticated regulatory network governing bacterial wilt tolerance. The genome-wide studies not only identified key players in the plant’s defense response but also laid the groundwork for targeted breeding programs aimed at enhancing the resilience of potato varieties to this devastating pathogen. The implications of this study are far-reaching, holding the potential to revolutionize potato breeding strategies. By understanding the genomic intricacies of bacterial wilt tolerance, researchers and breeders can now work hand in hand to develop potato varieties with enhanced resistance, ensuring global food security in the face of agricultural challenges.

Basmati rice, celebrated for its aromatic grains, stands at the center of an ambitious molecular mapping endeavor. The quest is to unravel the genetic underpinnings of grain dimension traits, an essential aspect of rice quality and market preference. Malik et al. deployed state-of-the-art molecular mapping techniques to pinpoint quantitative trait loci (QTLs) responsible for variations in grain length, width, and thickness. The results unveiled a complex genomic landscape, with multiple QTLs exerting influence over distinct grain dimensions. This newfound knowledge provides a valuable resource for rice breeders seeking to craft varieties with tailored grain characteristics. The implications of this study extend beyond the laboratories and into rice paddies globally. Armed with the genetic roadmap of grain dimension traits, breeders can now accelerate the development of Basmati rice varieties that not only meet but exceed consumer expectations, securing the prominence of this prized rice variety in international markets.

The flag leaf of bread wheat emerges as a focal point in the quest for optimizing grain yield. As the primary site for nutrient remobilization and grain filling, understanding the genetic drivers of flag leaf development is paramount for sustainable wheat production. This study done by Mehla et al. dives deep into the structural and functional nuances of candidate genes associated with different developmental stages of the flag leaf. Transcriptomic analyses coupled with functional genomics shed light on the orchestration of key physiological processes, including photosynthesis, nutrient transport, and senescence. The implications of this research reverberate across wheat fields, offering a nuanced understanding of how genetic factors influence grain yield. By unraveling the intricacies of flag leaf development, researchers pave the way for targeted genetic interventions, enhancing nutrient remobilization and ultimately boosting the yield potential of bread wheat.

Pepper, a staple in culinary landscapes worldwide, takes center stage in a genome-wide exploration of the OVATE FAMILY PROTEIN (OFP) gene family. This family of genes has emerged as a key regulator of plant development and stress responses. Luo et al. conducted an exhaustive analysis, unveiling multiple members of the *OFP* gene family in pepper. Each member exhibited unique expression patterns across different tissues and responded differentially to various stress conditions. Structural analyses provided insights into conserved motifs and domains, offering tantalizing clues about the functional roles of these genes. The implications of this study extend to the realms of stress-tolerant crop development. Understanding the diversity and functions of the *OFP* gene family in pepper provides researchers and breeders with molecular tools to fortify pepper varieties against environmental stresses, ensuring a resilient and productive crop.

Fusarium head blight (FHB), a scourge of wheat crops globally, prompts an intensive investigation into genetic sources of resistance. This study seeks to identify specific loci associated with resistance to FHB, offering a glimmer of hope to wheat farmers battling this devastating disease. Wu et al. undertook a comprehensive analysis, uncovering genetic sources of resistance and specific loci linked to Fusarium head blight resistance in wheat. Molecular markers tightly linked to resistance genes provide a powerful tool for breeders striving to develop FHB-resistant wheat varieties. The implications of this study resonate in wheat fields beset by FHB, providing a roadmap for developing resilient varieties. Armed with molecular markers, breeders can implement targeted selection strategies, fortifying wheat crops against the destructive impact of Fusarium head blight.

The architecture of maize leaves, particularly leaf angle, emerges as a critical determinant of light interception and photosynthetic efficiency. This study employs a combination of genome-wide association studies (GWAS) and meta-analysis to identify candidate loci influencing leaf angle in maize. Wu et al. successfully unearthed specific genomic loci associated with leaf angle variation in maize. These loci, acting as pivotal regulators of leaf architecture, hold the key to optimizing planting density and light capture in maize fields. The implications of this research unfold in maize fields worldwide, offering actionable insights for optimizing plant architecture. Crop management practices and breeding strategies can now be fine-tuned, harnessing the genetic determinants of leaf angle to enhance the overall productivity of maize crops.

Micronutrient deficiencies, particularly in iron (Fe) and zinc (Zn), pose a global health challenge. This study delves into the genetic basis of grain Fe and Zn content in wheat, particularly under drought and heat stress conditions. The genome-wide association study (GWAS) done by Devate et al. uncovered specific genomic regions linked to variations in grain Fe and Zn content under drought and heat stress. Understanding the genetic factors influencing nutrient content provides insights for biofortification strategies to enhance the nutritional value of wheat grains. The implications of this study transcend the field of agriculture, holding promise for addressing global malnutrition. The identification of genomic regions associated with grain Fe and Zn content offers a foundation for breeding wheat varieties with improved nutritional profiles, contributing to efforts to combat nutrient deficiencies.

Sugar beet, a vital sugar-producing crop, faces the challenge of drought stress impacting both yield and sugar content. This study employs a genome-wide association study (GWAS) to uncover the genetic factors contributing to drought tolerance traits during the seedling stage in sugar beet. Li et al. identified specific genomic regions associated with drought tolerance traits in sugar beet. Candidate genes within these regions provide valuable insights into the molecular mechanisms underlying adaptive responses to drought stress, laying the foundation for breeding resilient sugar beet varieties. The implications of this study extend to sugar beet fields grappling with water scarcity. By unraveling the genetic basis of drought tolerance, researchers empower breeders with the knowledge needed to develop sugar beet varieties capable of thriving in water-limited environments.

Drought stress poses a severe threat to global wheat production. This study explores the application of marker-assisted selection (MAS) to expedite the transfer of drought tolerance quantitative trait loci (QTLs) to elite wheat lines, offering a targeted approach to developing resilient varieties. Sunilkumar et al. successfully employed MAS to transfer identified QTLs for drought tolerance to a promising wheat line. This approach not only accelerates the breeding process but also showcases the potential of molecular markers in developing drought-tolerant wheat varieties. The implications of this research reverberate across wheat-growing regions facing water scarcity. The successful application of MAS provides a blueprint for breeders, offering a precision tool to fortify wheat varieties against the challenges posed by drought stress.

Subtropical regions grapple with the dual challenges of drought and heat stress, significantly impacting wheat yield. This study investigates the genotypic capacity of post-anthesis stem reserve mobilization in wheat, focusing on its role in sustaining yield under stress conditions. The research done by Gurumurthy et al. provides insights into genotypic variations in post-anthesis stem reserve mobilization among different wheat varieties. Understanding the capacity of stem reserves to contribute to grain yield under stress conditions offers valuable information for selecting resilient genotypes for subtropical environments. The implications of this research are particularly relevant for wheat cultivation in subtropical regions facing the brunt of climate change. By identifying wheat varieties with enhanced post-anthesis stem reserve mobilization, researchers and farmers can work towards sustaining grain yield even under challenging climatic conditions.

In conclusion, these studies collectively weave a narrative of profound advancements in plant genomics. Each study unravels layers of genetic complexity, offering insights that range from bolstering crop resilience against diseases to optimizing nutrient content and enhancing yield sustainability. The intricate tapestry of genetic codes governing diverse traits emerges as a dynamic landscape, ripe with possibilities for crop improvement. As researchers decipher the genetic mysteries embedded in plant genomes, the promise of translating these insights into resilient, high-yielding crops becomes increasingly tangible. These advancements not only pave the way for a more sustainable and secure global food supply but also exemplify the collaborative and interdisciplinary nature of contemporary plant science. The journey into plant genomics is an ongoing exploration, promising a future where crops are not just resilient but finely tuned to meet the evolving demands of a changing world.

